# Hydrogen Sulfide in Pharmacotherapy, Beyond the Hydrogen Sulfide-Donors

**DOI:** 10.3390/biom10020323

**Published:** 2020-02-18

**Authors:** Ewelina Zaorska, Lenka Tomasova, Dominik Koszelewski, Ryszard Ostaszewski, Marcin Ufnal

**Affiliations:** 1 Department of Experimental Physiology and Pathophysiology, Laboratory of Centre for Preclinical Research, Medical University of Warsaw, 02-091 Warsaw, Poland; ewelina.zaorska@gmail.com; 2 Institute of Clinical and Translational Research, Biomedical Research Center, Slovak Academy of Sciences, 84505 Bratislava, Slovakia; lennytomasova@gmail.com; 3 Institute of Organic Chemistry, Polish Academy of Sciences Kasprzaka 44/52, 01-224 Warsaw, Poland; medyk20@gmail.com (D.K.); ryszard.ostaszewski@icho.edu.pl (R.O.)

**Keywords:** hydrogen sulfide, H_2_S donors, H_2_S pro-drugs, sulfur-containing drugs, cardiovascular, neuromodulation, anticancer drug, anti-inflammatory agents

## Abstract

Hydrogen sulfide (H_2_S) is one of the important biological mediators involved in physiological and pathological processes in mammals. Recently developed H_2_S donors show promising effects against several pathological processes in preclinical and early clinical studies. For example, H_2_S donors have been found to be effective in the prevention of gastrointestinal ulcers during anti-inflammatory treatment. Notably, there are well-established medicines used for the treatment of a variety of diseases, whose chemical structure contains sulfur moieties and may release H_2_S. Hence, the therapeutic effect of these drugs may be partly the result of the release of H_2_S occurring during drug metabolism and/or the effect of these drugs on the production of endogenous hydrogen sulfide. In this work, we review data regarding sulfur drugs commonly used in clinical practice that can support the hypothesis about H_2_S-dependent pharmacotherapeutic effects of these drugs.

## 1. Hydrogen Sulfide in Physiology and Pharmacology

**Figure 1:**
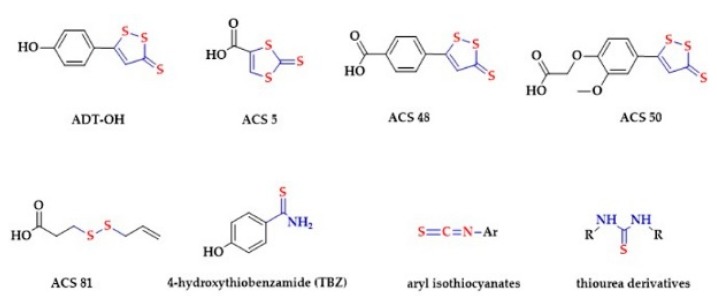
Examples of hydrogen sulfide (H_2_S)-releasing groups that can be coupled to existing pharmacologically active compounds.

Hydrogen sulfide (H_2_S) is produced by a variety of organisms, e.g., bacteria, fungi, plants and animals. First reports linking H_2_S with the smell of rotten eggs can be traced back in the 18th century (reviewed in [1]). Similarly, the toxic effects of H_2_S on mammals have been known over the centuries. The 1996 report from Abe and Kimura, suggesting the role of endogenously produced H_2_S in neuromodulation, started a new era in H_2_S research, and its role in biology and medicine [2]. Later, a number of important biological actions of H_2_S were described, including vasorelaxation [3], changes in brain neurotransmission [4,5,6], and the effect on neuronal K^+^ channel activity [7]. These effects are believed to be mediated by physiological concentrations of H_2_S. Therefore, H_2_S is now regarded as a third gaseous signaling molecule, next to nitric oxide (NO) and carbon monoxide (CO). In order to develop H_2_S-releasing donors, researchers started to modify chemical structures of well-described sulfide releasing agents, obtaining several H_2_S donors including Lawesson’s reagent and analogues [8], DTT (1,2-dithiole-3-thiones) derivatives like ADT-OH, ACS 5, ACS 48 and ACS 50 [9,10,11], diallyl disulfide (DADS) derivatives like ACS 81 [12], arylthioamides (TBZ) [13], aryl isothiocyanates [14] and thiourea derivatives [15] (Figure 1).

Interestingly, there are numerous well-established medicines which contain sulfur moieties. It can be assumed that drugs containing sulfur in their structure may release H_2_S or affect its endogenous production. The possibility of releasing H_2_S from drugs can enhance their biological activity and provide additional therapeutic benefits, but also generate some adverse effects. This paper reviews experimental and clinical data that may suggest that the pharmacological effects of several commonly used drugs may in part depend on the presence of sulfur groups and/or on the release of H_2_S from the active molecule.

### 1.1. H_2_S Production

The H_2_S gas is colorless and flammable. Free sulfide is a weak acid that dissociates in the body fluids with pH 7.4, the pKa1 = 6.8 and pKa2 > 12 at 37 °C to yield ~20% of H_2_S and ~80% of HS^–^ and negligible amounts of S^2-^ [16]. In cellular compartments the pH affects the relative proportion to total sulfide, from 90% of HS^–^ in the mitochondrial matrix (pH = 8) to over 90% of H_2_S in lysosomes (pH = 5). The lipophilic property enables a rapid diffusion of H_2_S through the lipid bilayer of cell membranes [17]. On the other hand, HS^–^ is not permeable and requires transporters in order to enter the cell [18,19].

H_2_S is produced in mammalian organisms by non-enzymatic and enzymatic pathways. Sulfate-reducing bacteria (SRB) colonize the gut and in the presence of an electron donor reduce sulfate to produce H_2_S [20]. In addition, erythrocytes are able to convert elemental sulfur to HS^-^ by non-enzymatic reduction [21]. H_2_S is generated in the tissues by cysteine metabolizing enzymes, cystathionine β-synthase (CBS), cystathionine γ-lyase (CSE) and 3-mercaptopyruvate sulfurtransferase (3-MST) in conjunction with cysteine aminotransferase (CAT). CBS and CSE are a part of the reverse transsulfuration pathway. The β-replacement reaction of homocysteine with serine is catalyzed by the CBS and generates cystathionine. CSE catalyzes the α,γ-elimination of cystathionine to cysteine, α-ketobutyrate and NH_3_. H_2_S is generated subsequently by the β-elimination reaction of cysteine catalyzed by either CBS or CSE. Alternatively, CSE catalyzes the conversion of cystine to thiocysteine, pyruvate and NH_3_, subsequently liberating H_2_S [22]. CAT catalyzes the conversion of cysteine to α-ketoglutarate, producing 3-mercaptopyruvate. 3-MST subsequently forms a persulfide on the enzyme, liberating H_2_S under reducing conditions [23]. CBS represents the main H_2_S-generating enzyme in the brain, whereas CSE dominates in the cardiovascular system [24,25]. The activity of 3-MST seems to be highest in the adrenal cortex [26]. The expression of the enzyme was also reported in erythrocytes [27]. All enzymes can be found in the lungs, liver, kidneys and gastrointestinal tract [28,29,30,31]. Regarding the sub-cellular distribution, CBS and CSE are cytosolic enzymes [32], whereas 3-MST is present mostly in the mitochondria [33]. However, translocation of these enzymes between compartments is possible under specific conditions [33,34]. In addition to cysteine metabolism, several other pathways of H_2_S biosynthesis were proposed, including the reduction of thioredoxin by catalase or thiosulfate by thiosulfate reductase [35,36]. Finally, gut bacteria express specific H_2_S-producing enzymes, namely cysteine desulfhydrase [37,38] and sulfite reductase [39].

### 1.2. H_2_S Excretion

The main route of elimination is the oxidation of H_2_S in the mitochondria. H_2_S is converted to thiosulfate and further oxidized to sulfate and excreted by the kidneys. The main enzymes involved in the elimination pathway are sulfide quinone oxidoreductase (SQR), persulfide dioxygenase (ETHE1), thiosulfate sulfurtransferase (TST) and sulfite oxidase (SO). Firstly, a SQR cysteine persulfide is formed. The sulfane can be further transferred to glutathione to form glutathione persulfide or to sulfite and form thiosulfate. The glutathione persulfide may be oxidized by ETHE1 and thiosulfate by TST to regenerate sulfite, which is oxidized by SO to sulfate [36,40,41]. Additionally, a part of H_2_S is exhaled or scavenged in the blood by methemoglobin to form sulfhemoglobin [42,43,44].

### 1.3. H_2_S Concentrations in Plasma and Tissues

The concentration of free H_2_S in plasma and tissues is in nanomolar range [45]. In contrast, very high concentrations of H_2_S are found in the large intestine [46,47,48]. This is because of enzymatic production by the intestinal tissue, and non-enzymatic and enzymatic production by gut microbiota. It has been found that colonic epithelial cells convert sulfide into thiosulfate more efficiently than other tissues [49,50]. Shen et al. reported that germ-free mice have lower systemic levels of H_2_S in plasma and various tissues, suggesting that gut microbiota regulates the systemic bioavailability and metabolism of H_2_S [51].

Free H_2_S may exist in bound form as sulfane sulfur or acid labile sulfur. Inorganic (H_2_S_n_) or organic (RS_n_H or RS_n_R) persulfides (*n* = 2) and polysulfides (*n* = 3–8) represent the sulfane sulfur store [52]. These species are formed either by direct interaction between H_2_S and oxidants (GSSG, NO) or by enzymatic oxidation. For instance, the persulfidation of 3-MST (3-MST-SSH) or SQR (SQR-SSH) can represent a source of organic persulfides [32,35]. In addition, 3-MST, super oxide dismutase (SOD) and catalase may oxidize H_2_S and form inorganic and organic per-/poly-sulfides [36,53,54,55,56]. Interestingly, catalase acts as sulfide-sulfur oxido-reductase, catalyzing both the H_2_S oxidation or the thiols reduction and H_2_S production [34]. Endogenous reductants subsequently liberate H_2_S from sulfane sulfur stores or the sulfane may be transported and transferred to other molecules to mediate sulfur signaling [52,56]. The acid labile sulfur is formed by the interaction between H_2_S and iron centers of proteins. However, the H_2_S release from the acid labile store requires low pH < 5.4 [57].

### 1.4. H_2_S Signaling

A number of cellular and molecular mechanisms of H_2_S actions have been proposed, including the interaction of H_2_S with several ion channels, enzymes regulating redox balance, the persulfidation or a direct interaction with heme proteins.

Increasing evidence suggests that physiological effects of H_2_S are linked with the persulfidation of the target protein residues [58,59]. The persulfidation is a crucial post-translational modification that regulates the function of the proteins. In order to form a cysteine persulfide, the oxidation of H_2_S to per-/poly-sulfide or the oxidation of the target cysteine to sulfenic acid or disulfide is needed [60]. Recently, the endogenous source of persulfides was identified in the mitochondria, namely the cysteinyl–tRNA synthetases, which incorporate cysteine persulfides into the proteins during translation. It was hypothesized that the cysteine persulfides may be released to cytosol in order to mediate further post-translational persulfidation of target proteins [61].

In addition, the interaction of H_2_S with metal centers of target proteins, particularly the interaction with heme proteins, was investigated thoroughly [62]. H_2_S may induce a covalent modification of heme, resulting in sulfheme formation [63]. Secondly, the oxidative detoxification of H_2_S by heme proteins results in the formation of polysulfides and thiosulfate [27]. For instance, the toxic effect of H_2_S is based on the inhibition of mitochondrial electron transport at cytochrome C oxidase [64,65,66]. H_2_S reversibly binds to the heme center of cytochrome C oxidase, thereby inhibiting the binding of oxygen, resulting in the shutdown of ATP generation [66,67]. On the other hand, low concentrations of H_2_S (≤1 µM) stimulate cellular energetic. The persulfidation of SQR is coupled with the transfer of electrons to coenzyme Q, thereby enhancing mitochondrial electron transport, resulting in higher ATP production [68,69].

H_2_S may also modulate the production and activity of other gasotransmitters. The persulfidation of endothelial NO-synthase (eNOS) Cys_433_ residue promotes the production of NO [70]. The persulfidation of Keap 1 Cys_151_ leads to the dissociation of the protein from Nrf2, subsequent translocation of Nrf2 into the nucleus, thereby promoting the heme oxygenase 1 (HO-1) induced CO production [71]. Similar to persulfidation, NO may modulate protein function via S-nitrosation. However, Wolhuter et al. reported that S-nitrosation is not a stable regulatory modification in the cells. They proposed that S-nitrosothiols are transient intermediates that react with thiols to form stable persulfides [72]. The direct interaction between H_2_S and NO results in the formation of biologically active nitrosopersulfide and polysulfides [73,74,75,76]. In addition, H_2_S may interact with other reactive species, e.g., oxygen, nitrogen, sulfur and selenium species. These species are produced by various cellular enzymes (NADPH oxidase, xanthine oxidase, uncoupled NOS) and their mutual interaction leads to the formation of numerous products, contributing mostly to the redox biology of the cell [77,78,79,80,81].

### 1.5. H_2_S in the Cardiovascular System

Vasodilation and blood pressure lowering induced by exogenous H_2_S salts and H_2_S donors have been reported by several groups [82,83,84,85,86,87,88,89,90,91,92]. The endogenous production of H_2_S by CSE was decreased in various types of hypertension, e.g., in spontaneously hypertensive rats, in rats with pulmonary hypertension and in women with pre-eclampsia, compared to healthy controls [24,93,94,95]. Moreover, the deletion of CSE in mice resulted in the development of hypertension and impaired endothelium-dependent vasorelaxation [24]. We have recently shown that, besides tissue enzymes, the gut microbiota-derived H_2_S may be involved in the development of hypertension [83,89,96]. In addition, H_2_S donors were found to relax corpus cavernosum and were tested for the treatment of erectile dysfunction [97,98,99,100].

The opening of ATP-sensitive potassium channels (K_ATP_) is believed to mediate the vasodilation induced by H_2_S donors [83,86,101]. Namely, the activation of the channel by persulfidation of the sulfonylurea receptor 1 (SUR1) Cys_6_ and Cys_26_ subunit [101,102]. Several studies confirm that the H_2_S-related vasodilation is associated with the promotion of NO synthesis (Cys_433_ eNOS persulfidation) and/or NO signalling (reviewed in [103]). For instance, the H_2_S/NO interaction product nitrosopersulfide mediates vasodilation and increases levels of cyclic guanosine monophosphate (cGMP) [73]. In addition, Stubbert et al. proposed a NO-independent mechanism of direct activation of protein kinase G (PKG) 1α by Cys_42_ persulfidation. They showed that transgenic knock-in mice, where Cys_42_ within PKG1α is replaced with redox-dead Ser, do not respond to H_2_S salt by blood pressure lowering [104].

The administration of H_2_S donors stimulates vascularization (reviewed in [105]) and silencing of 3-MST reduces cell growth, migration and network formation [106]. The activation of vascular endothelial growth factor (VEGF) or the inhibition of phosphatase and tensin homolog (PTEN) were proposed to mediate the pro-angiogenic actions of H_2_S. In detail, the direct reduction of Cys1024-Cys1045 within VEGF2 by H_2_S was reported [107]. In addition, the persulfidation of Cys68 and Cys755 specificity protein 1 (Sp 1) promoted the transcription of VEGF2 [108]. Greiner et al. observed the formation of PTEN Cys124 and Cys71 disulfide bond as a response to H_2_S salts [60].

### 1.6. H_2_S and the Immune System

Two major pathways regulate the inflammatory signaling in cells, namely the nuclear factor-κB (NF-κB) pathway and nuclear factor-erythroid 2-related factor 2 (Nrf2) pathway. Accumulating evidence suggests that H_2_S signaling promotes the Nrf2 signaling, thereby activating the antioxidant defense of the cell [71,109,110,111,112,113,114,115]. Nrf2 is sequestered by Kelch-like ECH-associated protein (Keap) 1 in the cytosol. The persulfidation of Keap 1 Cys_151_ leads to the dissociation of the protein from Nrf2 and subsequent translocation of Nrf2 to the nucleus, thus promoting the transcription of antioxidant response elements [71]. In addition, the persulfidation of Cys_38_ p65 subunit of NF-κB augments the binding to the ribosomal protein S3 (RPS3), thereby promoting the transcription of anti-apoptotic genes [116].

### 1.7. H_2_S in the Nervous System

Memory loss was reported in individuals exposed to toxic concentration of H_2_S [117]. In contrast, the breakthrough report from Abe and Kimura showed that micromolar H_2_S concentrations facilitate the induction of hippocampal long-term potentiation (LTP) [2]. The activation of *N-*methyl-D-aspartate (NMDA) receptor and the induction of Ca^2+^ influx by transient receptor potential ankyrin1 (TRPA1) channel opening were proposed to mediate the LTP induction by H_2_S donors [118]. In detail, the administration of sulfide salts and inorganic polysulfides led to the persulfidation of TRPA1 N-terminal cysteine residues and Ca^2+^ influx in astrocytes [118]. In addition, Kimura proposed that these sulfide species activate the NMDA receptor indirectly via the downstream TRPA1 signaling [119,120].

Decreased levels of H_2_S were reported in neurodegenerative disorders including Alzheimer disease and Parkinson disease in comparison to healthy controls [121,122,123,124]. Besides, lower expression of CSE was found in patients with Huntington disease [123]. On the other hand, a mutation of ETHE1 gene was found in ethylmalonyl encephalopathy patients resulting in the accumulation of H_2_S in the brain [125]. Several studies support the neuroprotective effects of H_2_S (reviewed in [118]). H_2_S may promote the glutathione (GSH) production via the activation of cystine/glutamate antiporter, cysteine transporter or glutamate cysteine lyase, and thus promote the antioxidative defense [126,127]. In addition, the opening of K_ATP_ and cystic fibrosis transmembrane conductance regulator Cl^-^ channels by H_2_S results in stabilizing of the neuronal plasma membrane [127]. The inactivation of neuroprotective ubiquitin E3 ligase of parkin plays a crucial role in the development of Parkinson disease. Vandiver et al. showed that persulfidation of parkin promotes the ubiquitin E3 ligase activity and thus mediates cytoprotection. Furthermore, they found that Parkinson’s patients have depleted persulfidated parkin in the brain [128].

### 1.8. Other Effects of H_2_S

A bell-shaped model characterizes the cellular effects of H_2_S. At lower concentrations, H_2_S promotes cell survival, whereas higher H_2_S concentrations can lead to cell death. The cytoprotective and anti-inflammatory properties of H_2_S are associated with faster dermal wound healing, mucosal defense and ulcer healing in the gastrointestinal system (reviewed in [129,130]). An improved clinical severity index of psoriasis was shown after the topical administration of H_2_S donor [131]. Furthermore, H_2_S-releasing derivatives of nonsteroidal anti-inflammatory drugs (HS-NSAIDs) reduced the gastric damage induced by the corresponding parent drugs (reviewed in [132]). H_2_S donors were also shown to relieve visceral pain [133,134,135] and an HS-trimebutine is now in Phase II clinical trials as an abdominal analgesic (NCT01926444). The role of H_2_S has also been investigated in the etiology of cancer and diabetes, however, the studies show contradictory results [136,137,138,139,140,141,142,143,144,145,146,147]

## 2. Sulfur-Drugs and Their Therapeutic Potential

Sulfur is essential to the life and growth of all organisms and plays a crucial role in the regulation of various biological processes in the human body. Sulfur can obtain oxidation states anywhere between −2 to +6 and represents one of the most chemically versatile elements. Generally, organo-sulfur compounds are organic compounds containing a carbon–sulfur bond. Many organo-sulfur compounds are sulfur equivalents of oxygen-containing organic compounds, for example, thioethers, thiols or thioesters. Therefore, sulfur-containing products can form a variety of molecular arrangements and exhibit diverse biological activities. The organo-sulfur compounds were already used as ointments with mild antiseptic effects in ancient times. The colloidal sulfur was regularly administered to patients suffering from rheumatoid arthritis. At present, the diversity of elements among approved pharmaceuticals reveal that sulfur is the fifth most used element after carbon, hydrogen, oxygen and nitrogen [148]. Sulfur-derived functional groups possess a variety of pharmacological properties and represent a useful tool for the development of new therapeutic agents. Sulfur moieties can be found in pharmaceuticals with various therapeutic applications, particularly in antihypertensive drugs, analgesics, antibacterial, anti-inflammatories, anticancer agents and many others.

### 2.1. Natural Products Containing Hydrogen Sulfide-Releasing Moieties

**Figure 2:**
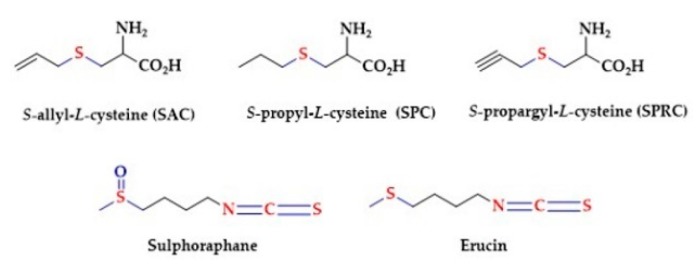
Naturally occurring H_2_S-donating compounds.

Natural products capable of releasing H_2_S have drawn a lot of attention [149]. Commonly isolated compounds from sulfur natural products are allyl-substituted polysulfides (mainly in form di-, trisulfides and/or tetrasulfides) [79,150,151,152,153]. The garlic-derived sulfur compounds like the diallyl disulfide (DADS) require the presence of reduced glutathione to release H_2_S. H_2_S generation relies on nucleophilic substitution of GSH at the a-carbon of the allyl substituent to form an allyl perthiol, which further undergoes a thiol/disulfide exchange to release H_2_S. Similarly, red blood cells released H_2_S rapidly from DADS under anoxic conditions and in the presence of glutathione [154]. The health benefits of garlic have been postulated for thousands of years and several studies demonstrated the positive impact of garlic on the cardio-vascular system. This includes lowering of arterial blood pressure, the reduction of blood cholesterol and platelet aggregation, and the reduction of oxidative stress. It was suggested that *S-*allyl-*l-*cysteine (SAC) is a potential source of H_2_S and is responsible for the cardioprotective effects of garlic. Other garlic-derived compounds are *S-*propyl*-L-*cysteine (SPC) and *S-*propargyl-*l-*cysteine (SPRC) [155,156,157]. In addition to garlic, there are many other natural products containing functional groups that can be considered as potential H_2_S donors, for example Sulphoraphane and Erucin (Figure 2) [158,159].

Sulforaphane is sulfur-organic molecule from the group of isothiocyanates. Sulphoraphane occurs in cruciferous vegetables and its highest concentrations are found in broccoli sprouts. Sulphoraphane has been postulated to exert anticancer property, to suppress the proliferation of prostate cancer cells and to enhance the expression of CBS and CSE [160]. It has also been postulated that the consumption of Broccoli sprouts, containing Sulforaphane, reduces nephropathy and vascular complications [158].

### 2.2. Sulfur Amino Acids

**Figure 3:**
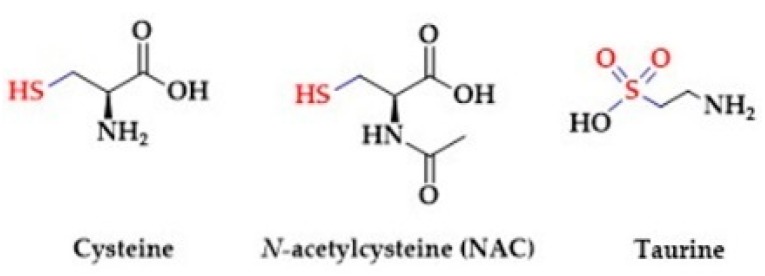
The sulfur-containing amino acids.

Several studies confirm that dietary sulfur amino acids, cysteine and taurine (Figure 3), have beneficial effects on human health [161].

**Figure 4:**
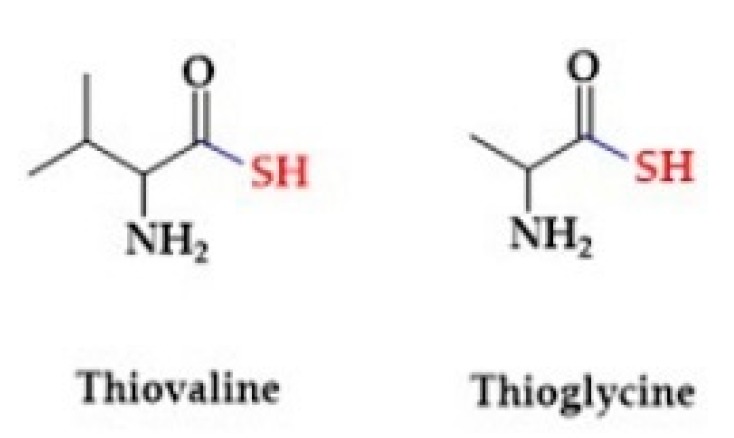
The structures of selected thiol amino acids.

Sulfur amino acids participate in the synthesis of essential bio-molecules like antioxidants, vitamins and co-factors (thiamine, lipoic acid, biotin, coenzyme A). Giannis et al. showed that thiol amino acids are potential H_2_S donors [162]. They observed the release of H_2_S from thioglycine and thiovaline (Figure 4) in the presence of bicarbonate. In addition, both sulfur amino acids promoted the cGMP formation and relaxation of mouse aortic rings [163].

#### 2.2.1. Cysteine

The chemical structure of cysteine contains a nucleophilic thiol (-SH) (Figure 3) that may be readily oxidized, thus mediating biological activity of the cells. The thiol group enables direct scavenging of free radicals or the regeneration of oxidized molecules to their reduced states. Furthermore, cysteine serves as a substrate for the production of glutathione and H_2_S. Cysteine residues incorporated within proteins play a key role in the regulation of structural and functional properties of proteins. Particularly, the formation of cysteine disulfides and persulfidation of cysteine residues (described in section *Signaling*) are crucial post-translational modifications. Cysteine is endogenously produced from an essential amino acid methionine. In detail, the demethylation of methionine results in the formation of *S-*Adenosyl-*l-*homocysteine (SAH), which is subsequently hydrolyzed to homocysteine. Homocysteine enters the transulfuration pathway to produce cysteine by CBS and CSE. Accumulating evidence suggests that cysteine plays a key role in the maintenance of mammalian homeostasis [164,165,166,167,168]. However, due to its unstable nature cysteine is not suitable for clinical use. *N-*acetylcysteine (NAC) has been used instead as a nutritional supplement over the years. Several reports confirm that administration of NAC prevented the development of hypertension in rodents and humans. In addition, NAC attenuated the hypertensive-related complications, namely increased nitric oxide bioavailability, improved renal function and attenuated the development of insulin resistance. In addition, the antioxidant properties of NAC are used to prevent the development of neurodegenerative disorders, inflammatory bowel disease or to treat paracetamol-induced poisoning [169,170].

#### 2.2.2. Taurine

Taurine (2-aminoethanesulfonic acid) is one of the few naturally occurring sulfonic acids -SO_3_H (Figure 3). It is endogenously produced via cysteine sulfinic acid pathway or acquired by diet [171,172]. In detail, the thiol moiety of cysteine is oxidized by cysteine dioxygenase to sulfinic acid. Further decarboxylation by sulfinoalanine decarboxylase forms hypotaurine, which is subsequently oxidized to taurine by hypotaurine dehydrogenase. Taurine is abundant in the brain, retina, skeletal muscle and liver of mammals. The transport of taurine through plasma membranes is mediated via transporters: SLC6A6 (TauT) and SLC36A1 (PAT1) [173]. Taurine is an important substrate for microbial production of H_2_S. Taurine is used by known intestinal microbe *Bilophila wadsworthia* as an electron acceptor for anaerobic respiration. This pathway results in sulfite production, which is subsequently converted to H_2_S [174]. Similar to cysteine, blood pressure lowering and antioxidative effects were reported after taurine supplementation [175,176,177,178,179,180]. Moreover, the development of hypertension was accelerated in taurine-deficient rats [181]. Taurine does not incorporate into proteins and the biochemical nature of its actions is not clear. Interestingly, the antihypertensive effect of taurine was associated with increased levels of H_2_S in the plasma of prehypertensive patients. Taurine upregulates the expression of H_2_S-synthesizing enzymes CBS and CSE, and thereby contributes to increasing the level of endogenous H_2_S [182].

### 2.3. Antihypertensive Drugs

Hypertension is a leading cause of morbidity and mortality worldwide. Numerous antihypertensive drug classes were developed, e.g., renin–angiotensin–aldosterone system (RAAS) inhibitors, calcium channel blockers, beta-blockers and diuretics. The RAAS is a key regulator of blood volume and systemic vascular resistance. The decrease of systemic blood pressure leads to the release of renin by the kidneys, thus stimulating the formation of angiotensin, which in turn promotes the release of aldosterone from the adrenal cortex, resulting in sodium and water retention in the kidney [183,184]. To date, over 20 compounds targeting the RAAS have been introduced, and some of them possess a sulfur moiety.

**Figure 5:**
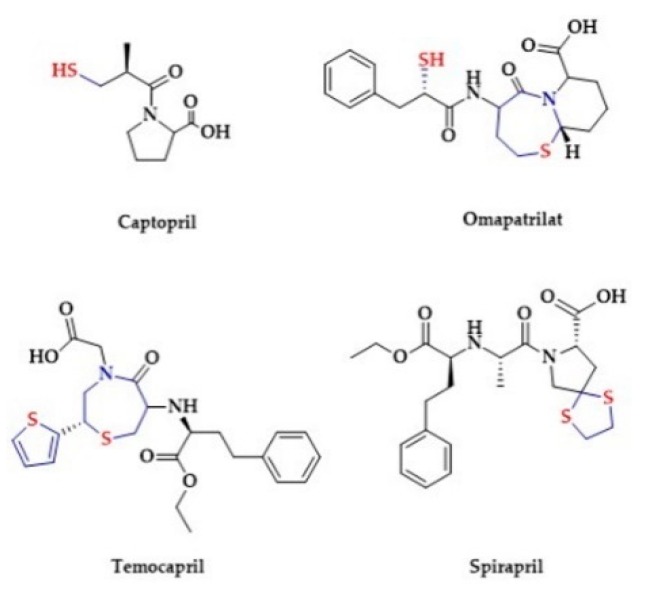
The structures of selected ACE inhibitors.

The group of angiotensin-converting enzyme inhibitors (ACE-I) is a cornerstone of antihypertensive treatment [185,186]. The first ACE-I, i.e., Captopril, was patented and approved for clinical use in the 1980 [187]. The chemical structure of Captopril contains a thiol. In the plasma Captopril forms its disulfide or reacts with cysteine and glutathione to form mixed disulfides, thus representing a sulfane sulfur source (Figure 5).

**Figure 6:**
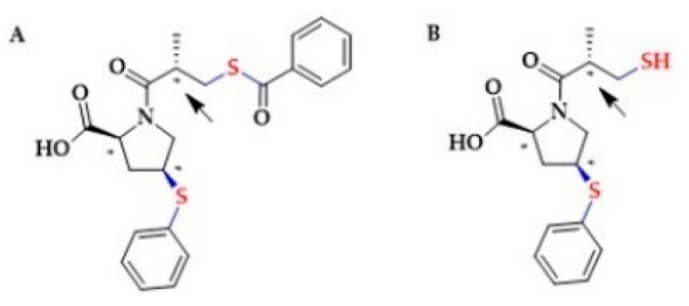
The structures of (**A**) Zofenopril and (**B**) Zofenoprilat. The asterisks denote the S configurations at the chiral centers. The arrow points to the only carbon atom whose configuration leads to R-Zofenoprilat.

However, the possible involvement of sulfide signaling in the Captopril-dependent effects remains unclear. Besides Captopril, Lisinopril is also administered as an active drug. Other ACE-I inhibitors are pro-drugs, undergoing hydrolysis in the liver to active forms containing a hydroxyl group [188]. Zofenopril, an ACE-I inhibitor approved for medical use in 2000, undergoes hydrolysis and forms an active metabolite Zofenoprilat containing a thiol group (Figure 6). Several studies confirmed that Zofenopril administration increases the levels of H_2_S-metabolites in the plasma of mice and pigs [188]. Pro-angiogenic, anti-inflammatory and anti-apoptotic actions of Zofenopril were reported in association with H_2_S release [189,190,191,192,193].

**Figure 7:**
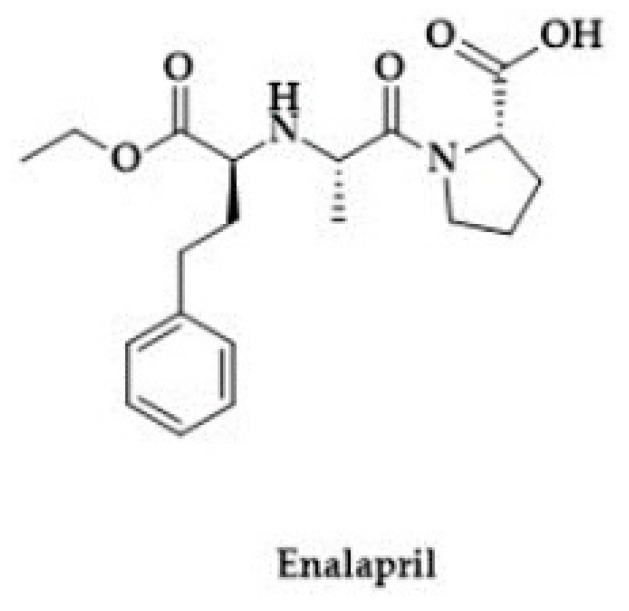
The structure of Enalapril.

In addition, Bucci et al. reported that Zofenopril improved vascular function in a model of spontaneous hypertension, which was associated with H_2_S release and was dependent on the inhibition of ACE. Namely, S-Zofenoprilat, the active diasteroisomer, as well as the inactive R-Zofenoprilat, restored vascular response of hypertensive rats (Figure 6). On the other hand, Enalapril, a non-thiol ACE inhibitor, failed to improve the vascular function (Figure 7) [192].

**Figure 8:**
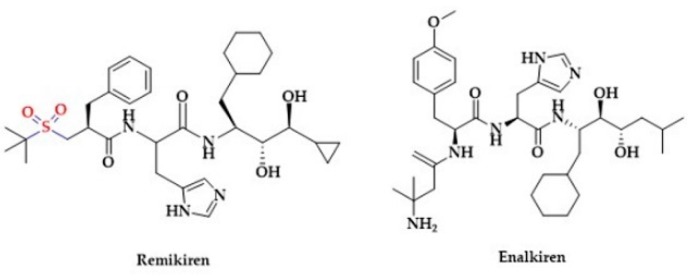
The structures of renin inhibitors Remikiren and Enalkiren.

Spirapril and Temocapril are hydroxyl-based ACE inhibitors, administered in the pro-drug esterified form. The chemical structure of these drugs contains cyclic sulfur moieties (Figure 5). Spirapril contains a sulfur atom in a dithioketal ring. Temocapril contains two sulfur atoms, one in the thiophene ring and the other in thiazepine ring [193]. In 2003, an experimental ACE inhibitor Omapatrilat was introduced. Omapatrilat contains a hydroxyl group as well as a thiol group and another sulfur atom in a thiazepine ring (Figure 5). It can simultaneously inhibit ACE and neutral endopeptidase (NEP). Interestingly, the ACE inhibition by Omapatrilat is longer in comparison to Enalapril [194]. Another sulfur-based drug is Remikiren, a direct renin inhibitor containing a sulfonyl moiety in its structure (Figure 8) [195].

**Figure 9:**
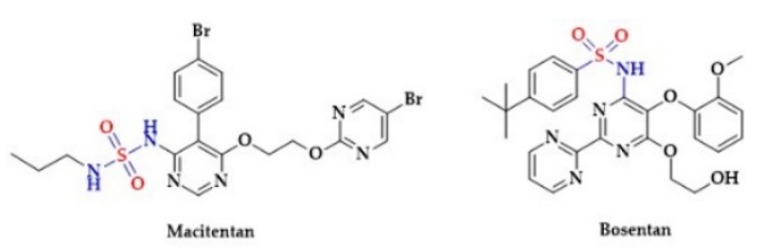
The structures of selected endothelin receptor antagonists.

Notably, a clinical study showed a greater potency of Remikiren to lower blood pressure in comparison to a non-sulfur renin inhibitor Enalkiren [196]. Several other antihypertensive drugs possess a sulfonic group in their structure. For instance, endothelin receptor antagonists Macitentan and Bosentan contain sulfones in their structure (Figure 9) [197,198].

**Figure 10:**
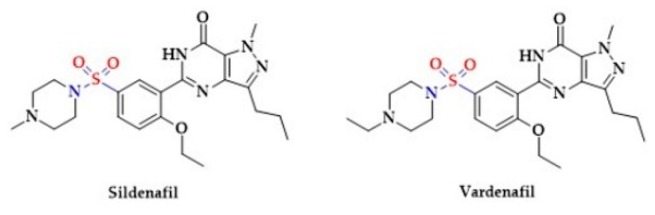
The structures of selected inhibitors of phosphodiesterase activity.

Moreover, the phosphodiesterase inhibitors, Vardenafil [199,200,201,202] and Sildenafil [199,203,204], used for the treatment of erectile dysfunction, are sulfonic acids (Figure 10).

**Figure 11:**
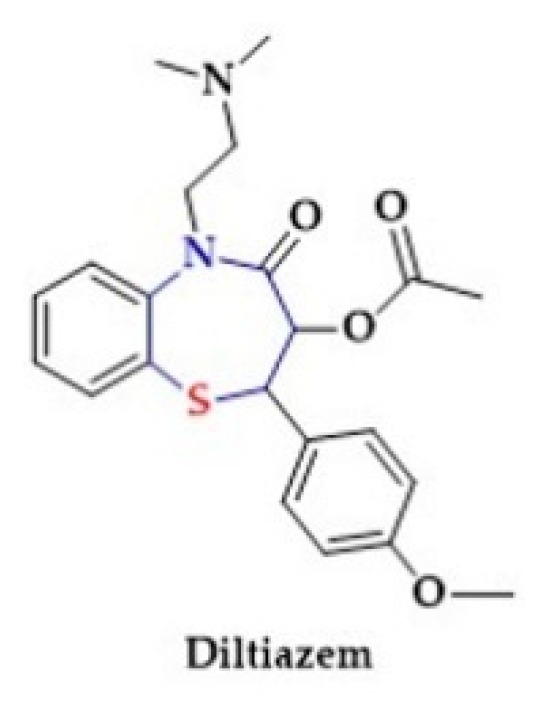
The structure of calcium-channel blocker (Diltiazem).

In addition, the calcium-channel blocker Diltiazem contains a thiazepine ring (Figure 11) [205].

### 2.4. Central Nervous System Agents

Numerous studies show that H_2_S exerts a number of biological actions in the Central Nervous System (CNS), including anti-inflammatory, anti-oxidant, anti-apoptotic, and neuroprotective effects [206].

**Figure 12:**
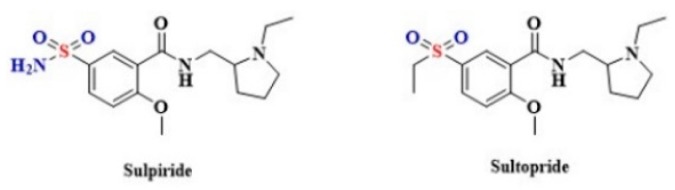
The structures of Sulpiride and Sultopride.

Despite the potentially beneficial effect of H_2_S on cellular functions, an excessive amount of H_2_S and polysulfides may impair brain functions in what is referred to as the so-called “sulfide stress” [207]. Sulfide stress is characterized by an increase in H_2_S/polysulfide production as a result of elevated levels of 3-MST enzyme. This may result from an inflammatory/oxidative insult to the brain. There is some evidence that the H_2_S/polysulfide production system is upregulated in schizophrenia. A more detailed explanation of the role of sulfide stress in the development of schizophrenia may give a new direction to develop a more effective treatment for this disorder [208]. However, it is worth stressing that several sulfur-based drugs are used in the treatment of schizophrenia, including Sulpiride and Sultopride. These medicines contain a sulfonamide group that is *S-*linked to a benzene ring (Figure 12) [209,210,211]. Whether the use of sulfuric drugs may affect the course of schizophrenia by modulating the endogenous H_2_S levels is unknown.

**Figure 13:**
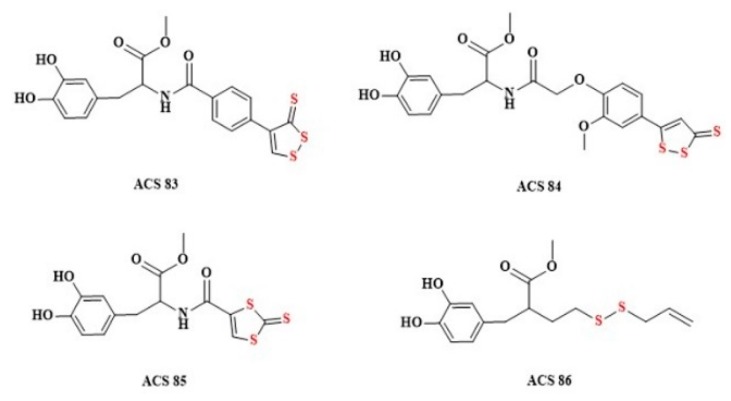
The structures of the H_2_S-releasing L-DOPA derivatives (ACS83, ACS84, ACS85, and ACS86).

Parkinson’s disease is a neurodegenerative disorder caused by progressive loss of dopaminergic neurons in the substantia nigra. The most widely used therapy is Levodopa (L-DOPA), but it does not stop disease progression [212]. Numerous studies indicate that the endogenous H_2_S levels are markedly reduced in various Parkinson’s disease models. Xue et al. showed that NaHS treatment reduces the loss of substantia nigra neurons and slows the development of motor dysfunction in animal models [213]. Other groups also found that intraperitoneal injection of NaHS (as H_2_S donor) and the inhalation of H_2_S exerted protective effects in animal models of Parkinson’s disease [214]. Based on these reports, it was stated that the combination of L-DOPA and H_2_S may have a potential therapeutic value. Lee at al. have developed four L-DOPA hybrids based on coupling L-DOPA to different hydrogen sulfide-donating compounds: ACS 48, ACS 50, ACS 5 and ACS 8 (Figure 13). H_2_S donor structures present in L-DOPA hybrids release hydrogen sulfide by hydrolysis.

After intravenous administration of H_2_S-releasing L-DOPA derivatives (Figure 13) a large increase in dopamine and glutathione has been observed in intracerebral fluid [215].

### 2.5. Dithiolethiones and Their NSAID Hybrids

**Figure 14:**
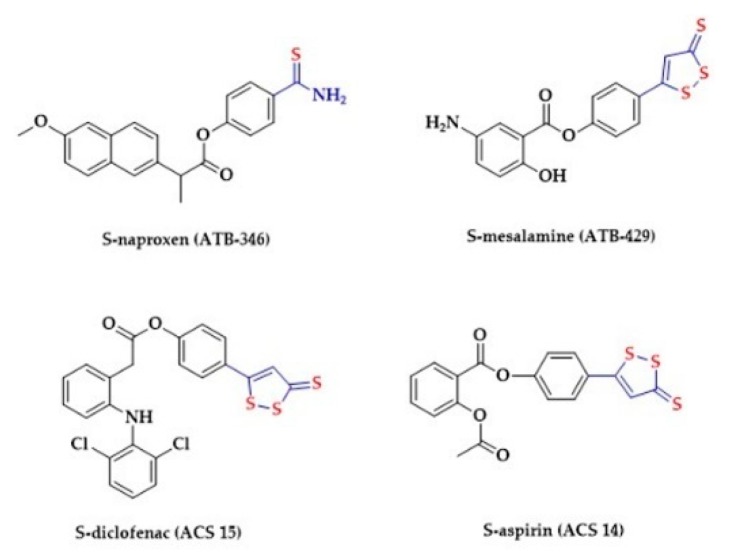
The structures of selected HS-NSAIDs.

NSAIDs have high efficacy in reducing pain and inflammation. The NSAIDs act by the inhibition of cyclooxygenases (COXs). Traditional NSAIDs are non-specific inhibitors of both COX-1 and COX-2. Adverse effects of NSAIDs on the gastrointestinal tract are associated with the reduction of prostaglandin synthesis due to the inhibition of COX-1. Numerous studies showed that H_2_S may reduce adverse effects of NSAIDs in the gastrointestinal tract [216,217]. 1,2-Dithiole-3-thiones (DTTs), anethole trithione (ADT) and the phenol derivative of ADT (ADT-OH) belong to the family of hydrolysis-triggered H_2_S donors (Figure 14). A rapid generation of H_2_S from DTT derivates was observed in the presence of mitochondria [218]. They are commonly used in the design of HS-NSAIDs (hydrogen sulfide-releasing non-steroidal anti-inflammatory drugs) [217,218]. Sparatore et al. synthesized a S-aspirin (ACS 14) and compared the gastric damages caused by ACS 14 and aspirin in rats (Figure 14) [219]. ACS 14 protected the gastric mucosa through increased H_2_S/glutathione production, HO-1 activation and isoprostane suppression. S-diclofenac (ACS 15) has also been studied. This drug showed increased anti-inflammatory activity compared to diclofenac in several models [220,221]. Another hybrid drug, S-mesalamine (ATB-429), has been well characterized in animal models of Crohn’s disease and ulcerative colitis and has turned out to be more effective than mesalamine [222]. Similarly, S-naproxen (ATB-346) has been found to cause less gastric damage than its parent drug [223]. Chattopadhyay et al. evaluated the effects of four different HS-NSAIDs on the growth of different human cancer cell lines. All tested HS-NSAIDs effectively inhibited the growth of cancer cells [224].

### 2.6. The Coxibs, Selective Inhibitors of Cyclooxygenase-2 (COX-2)

**Figure 15:**
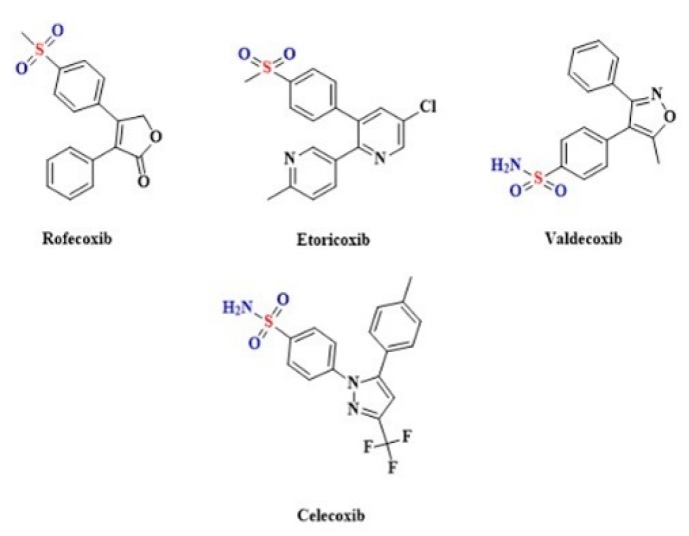
The structures of selective COX-2 inhibitors.

The Coxibs belong to the group of anti-inflammatory drugs that are selective inhibitors of COX-2 [225,226]. Celecoxib, Rofecoxib [227], Etoricoxib [228] and Valdecoxib [229] contain a sulfonamide group that is S-linked to a benzene ring (Figure 15).

Treatment with selective COX-2 inhibitors such as Celecoxib seems to produce fewer side effects in comparison with non-selective NSAIDs [230,231]. Szabó et al. synthesized a series of Celecoxib derivatives with various substituents on the benzenesulfonamide moiety. The gastrointestinal adverse reaction profile was more favorable compared to the parent drug [232]. Celecoxib is often used to counteract the multiple side effects of Cyclosporin A (CsA), an immunosuppressant drug used in the treatment of inflammatory diseases of autoimmune origin [233,234,235]. H_2_S was shown to prevent the CsA-induced vasomotor alteration and nephrotoxicity [236,237]. In addition, Helmy et al. confirmed that upregulation of CSE/H_2_S pathway underlies the capacity of Celecoxib to compromise the hypertensive and renal insult caused by CsA in rats [238].

### 2.7. Thiourea Derivatives As Antithyroid and Anesthetics Drugs

**Figure 16:**
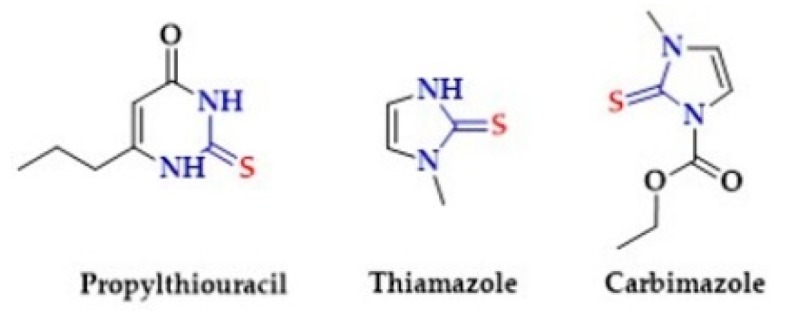
The structures of Propylthiouracil, Thiamazole and Carbimazole.

The antithyroid activity of thiourea and its derivatives has been confirmed in numerous studies. Thyreostatics containing thiourea in a cyclic form are Propylthiouracil, Thiamazole and Carbimazole (Figure 16).

Propylthiouracil inhibits the synthesis of thyroxine and inhibits conversion of thyroxine to triiodothyronine. Thiamazole (other name Methimazole) may directly inhibit thyroid peroxidase or directly inhibit thyroglobulin, hence reducing the production of the thyroid hormones T3 and T4 (thyroxine) [239,240]. Carbimazole is a pro-drug which is converted to the active form, methimazole [241]. In our work from 2018, we proved that compounds based on thiourea can act as controlled hydrolysis-based H_2_S donors [15]. In turn, numerous studies indicate an association between thyroid hormone (TH) level and H_2_S level [242,243].

**Figure 17:**
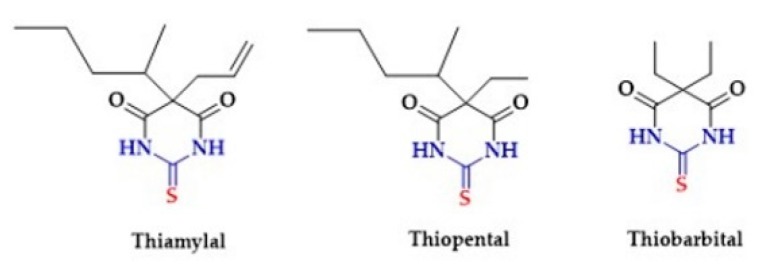
The structures of Thiamylal, Thiopental and Thiobarbital.

Another group of drugs containing thiourea moiety in the structure are barbiturates. Barbiturates act as CNS depressants. They are also used as anxiolytics, hypnotics, and anticonvulsants. The examples of sulfur-containing barbiturates are Thiamylal, Thiopental and Thiobarbital (Figure 17). Both Thiamylal, and Thiopental are used for short-term anesthesia and short surgical procedures associated with minimal painful stimuli [244,245]. Thiobarbital has sedative effects [246].

### 2.8. Other Drugs

**Figure 18:**
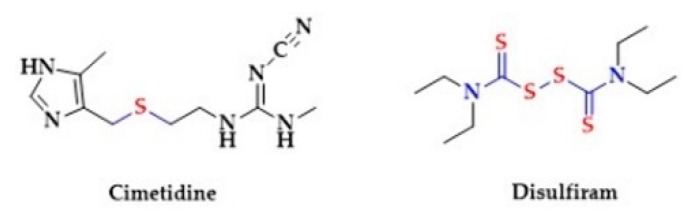
The structures of Cimetidine and Disulfiram.

Disulfiram in chemical terms is tetraethylthiuram disulfide. Thiuram disulfides are a class of organo-sulfur compounds with the formula (R_2_NCSS)_2_ (Figure 18).

There are two dithiocarbamate subunits which are linked by an S−S bond in the chemical structure of Disulfiram. This drug is used for the treatment of alcohol dependence [247]. It belongs to a group of aldehyde dehydrogenase inhibitors that increase the blood level of acetaldehyde after the ingestion of ethanol. The disulfiram–ethanol reaction (DER) is the cause of highly unpleasant symptoms referred to as “acetaldehyde syndrome,” including flushing, systemic vasodilation, respiratory difficulties, nausea and hypotension. The latter is one of the most common and potentially life-threatening side effects of the drug. The observed blood pressure-lowering effect has been attributed to the vasodilatory action of acetaldehyde [248].

Cimetidine, a histamine receptor blocker, contains a sulfur moiety in the form of thioether (Figure 18). Thioethers are sulfuric ether analogues with the general formula R−S−R. The effects of Cimetidine include reduction of gastric acid secretion and reduction in gastric volume and acidity. Interestingly, a common side-effect of Cimetidine is hypotension. It has been reported, that intravenous administration of Cimetidine induces a short-lasting (5–15 min) hypotension in anaesthetized rats due to arterial vasodilatation. Notably, the pretreatment with diphenhydramine, an antihistamine agent, did not reduce the hypotensive effect. This suggests no involvement of histamine receptors in the hypotensive action of cimetidine. It may be speculated that the release of H_2_S from the thioether moiety may be responsible for the cimetidine-induced hypotension [249].

**Figure 19:**
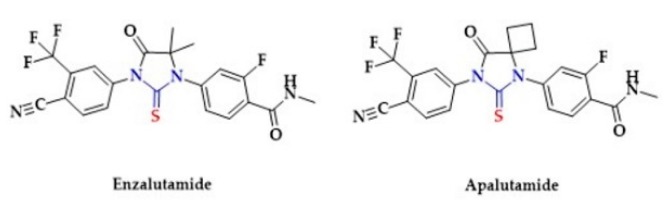
The structures of selected androgen receptor (AR) antagonists.

Several studies suggest that H_2_S may regulate cancer cell growth and tumor progression and that the expression of CSE and CBS is reduced in antiandrogen-resistant prostate cancer cells. Additionally, in antiandrogen-resistant prostate cancer cells, lower levels of endogenous H_2_S were found [250,251]. Interestingly, Enzalutamide [252,253] and Apalutamide [254], the androgen receptor antagonists that are used in the prostate cancer treatment, are *N, N-*disubstituted thiourea derivatives (Figure 19). The thiourea moiety presence in the structure of Enzalutamide and Apalutamide may release H_2_S and strengthen their androgen receptor antagonist properties. Hydrolysis is the mechanism of H_2_S generation from thiourea derivatives. [15].

## 3. Perspectives and Limitations

Accumulating evidence suggests that H_2_S contributes to the regulation of essential biological processes in mammals. In spite of the significant progress in the field of developing H_2_S donors, there is still a lack of compounds that would meet all requirements for the ideal H_2_S donor in clinical studies. Notably, there are a number of commonly used drugs containing sulfur moieties, which have been found to release H_2_S ex vivo, and some of them in vivo. This may significantly contribute to pharmacokinetics and pharmacodynamics of those drugs. Nevertheless, there are significant gaps in our knowledge that hinder clinical use of H_2_S donors. A list of questions to be answered includes, but is not limited to, the following: (i) What are therapeutic vs. toxic concentrations of H_2_S and its products? (ii) What are the mechanisms of H_2_S release from the drug? (iii) How to deliver H_2_S chronically in vivo at a constant rate? (iv) How to monitor plasma concentration of H_2_S and its products? (v) What are the mechanisms of H_2_S action?

## 4. Conclusions

There are numerous well-established medicines containing sulfur moieties that release H_2_S ex vivo and may release H_2_S in vivo. Thus, the sulfur moieties present in the drug structure may function as an H_2_S donor and/or affect endogenous H_2_S metabolism. Further research is needed to clarify whether the released H_2_S may contribute to the therapeutic effect of these drugs, and, if so, which of the mechanisms is dominant. If it is true, the addition of sulfur moieties may significantly affect the pharmacotherapeutic profile of parent drugs.
